# Asset vulnerability analytical framework and systems thinking as a twin methodology for highlighting factors that undermine efficient food production

**DOI:** 10.4102/jamba.v11i1.597

**Published:** 2019-04-18

**Authors:** Eromose E. Ebhuoma, Mulala D. Simatele, Henry B. Tantoh, Felix K. Donkor

**Affiliations:** 1 School of Geography, Archaeology and Environmental Studies, University of the Witwatersrand, Johannesburg, South Africa; 2 Center in Water Research and Development, University of the Witwatersrand, Johannesburg, South Africa

## Abstract

Food production in developing countries has been highly susceptible to both climatic and non-climatic stressors. To identify the factors that prevent the rural poor from producing food efficiently, various participatory methodologies have been utilised. However, most methodologies have implicitly illustrated how vulnerable the livelihood activities of the poor are from an asset-based perspective. As assets give people the capability to thrive, we make a case for the asset vulnerability analytical framework (AVAF) and systems thinking (ST) as an integrated methodological framework. Data for this study were obtained from the rural Delta State of Nigeria through the principles and traditions of participatory research, which include Venn (or institutional) diagrams, transect walks, brainstorming, community risk mapping and historical timelines. Findings indicate that the AVAF, on the one hand, will make it relatively easier for development practitioners to effectively identify the factors that undermine the poor’s ability to maximise their livelihood assets during food production. The ST, on the other hand, will enable development practitioners to visualise the long-term consequences of the continued inability of the poor to maximise their livelihood assets. This article argues that the utilisation of both AVAF and ST will simplify the complex challenges of decision-making. This, in turn, will facilitate the implementation of appropriate policy interventions to protect the crucial assets necessary for the rural poor to produce their food efficiently and sustainably.

**Keywords:** asset vulnerability analytical framework; systems thinking; subsistence farmers; Delta State; Nigeria.

## Introduction

Globally, climate change has been observed to escalate to unprecedented levels especially in the last two decades (Moser 2014; Perez et al. 2015). Effectively and efficiently tackling issues of climate change, as Lazarus (2009) puts forward, is one of the most complex problems that the world will have to contend with. This is arguably the case in sub-Saharan Africa (SSA), where low adaptive capacity, weak institutions and high levels of poverty, among others, *vis-à-vis* increasing climate change have combined to undermine effective food production (Conway & Schipper 2011; Perez et al. 2015). Consequently, studies have been conducted using various participatory methodologies to ascertain how vulnerable the agricultural practices of the rural poor in SSA are to both climatic (rising temperatures and erratic rainfall patterns) and non-climatic (e.g. ill-health and over-exploitation of natural resources in farming communities) stressors (Mavhura 2017; Mupakati & Tanyanyiwa 2017).

Participatory methodologies, as observed by Chambers (1994), have been effective in illuminating both the climatic and non-climatic stressors confronting vulnerable people. This is because it provides a fertile platform for individuals to explicitly share their experiences regarding the ways in which their livelihood practices have been adversely affected by both climatic and non-climatic stressors. Literature suggests that the most utilised participatory methodologies employed by researchers and development practitioners at grassroots level within the context of SSA include participatory impact assessments (PIAs), vulnerability mapping (VM), participatory vulnerability assessment (PVA), community-wide vulnerability and capacity assessment (CVCA), climate change vulnerability resilience (CCVR) and participatory rural appraisal (PRA) (Chambers 1994; Ibarraran, Malone & Brenkert 2010; Kuban & MacKenzie-Carey 2001; Leurs 1996; Preston, Yuen & Westaway 2011; Shahid & Behrawan 2008). Despite the aforementioned methodologies being utilised to a great degree of success, Moser (2011) contends that they have either neglected or only implicitly illustrated how vulnerable the poor are from an asset-based perspective (see Table 1).

**TABLE 1:** Participatory methodologies employed in conducting vulnerability assessments.

Methods	Main users	Key objectives	Focus on assets	References
Vulnerability mapping (VM)	Emergency or relief development institutions, for example, Tearfund and researchers	Analysis and mapping of vulnerabilities to identify strategies to reduce both climate- and non-climate-related risks	While it focuses on the asset portfolio of individuals and households, it is not detailed in explaining the dynamics and complexities of factors that facilitate the erosion of the aforementioned assets	Simatele (2009)
Climate change vulnerability resilience (CCVR)	IDS, IIED, Tyndall Research Center, Practical action	Increasing the ability of communities to withstand and recover from climate-change-related external shocks and stresses with an emphasis on economic well-being, stability of a community, social and political factors, institutional capacity, global interconnectivity and natural resource dependence	Assets addressed implicitly as approach attaches significance to governance quality at municipal and local levels	Simatele (2009)
Participatory impact assessment (PIA)	Development institutions, NGOs, CBOs and researchers	Identifying interventional measures and action plans	Places more emphasis on communal, rather than individual assets, as donors are concerned with measuring the impact, their interventional strategies will have in cushioning generic challenges	Simatele (2009)
Participatory rural appraisal (PRA)	Development institutions, NGOs, CBOs and researchers	Focuses on local people’s experiences of the ways climatic and non-climatic related risks impact on their lives and livelihoods	Places more emphasis on communal, rather than individual assets	Chambers (1994); Simatele (2009)
Participatory vulnerability assessment (PVA)	Emergency or relief institutions, for example, ActionAid International	Analysis and mapping of vulnerabilities to identify strategies to reduce both climate- and non-climate-related risks	Focuses both on communal and individual assets, but trivialises the individual assets that do not affect the majority of vulnerable participants	Simatele (2009)
Community-wide vulnerability and capacity assessment (CVCA)	Emergency or relief institutions, for example, Red Cross Society, NGOs and CBOs	Analysis and mapping of vulnerabilities to identify strategies to reduce both climate- and non-climate-related risks	Focuses both on communal and individual assets, but trivialises the individual assets that do not affect the majority of vulnerable participants	Kuban and Mackenzie-Carey (2001); Simatele (2009)

*Source*: Adapted from Moser, C., 2011, *A conceptual and operational framework for pro-poor asset adaptation to urban climate change*, viewed 24 October 2016, from https://pdfs.semanticscholar.org/04b1/bcdba119831cd40f0dde1073a5e9dd32a284.pdf.

IDS, Institute of Development Studies; IIED, International Institute for Environment and Development; NGOs, Non-Governmental Organisations; CBOs, Community-Based Organisations.

The focus on assets, as Moser and Stein (2011:4) argue, is crucial because of the widespread recognition that poverty encompasses a multiplicity of ‘deprivation that includes lack of capabilities, assets and entitlements’. Assets ‘are not simply resources people use to obtain a livelihood; they also give people the capability to be and to act’ (Bebbington 1999:2022). Therefore, the more assets an individual has or has easy access to, the less vulnerable the individual is likely going to be and vice versa (Moser & Satterthwaite 2008). Thus, the utilisation of the asset vulnerability analytical framework (AVAF) as a methodological approach to conduct weather-related vulnerability assessments will explicitly highlight the factors that impede the maximisation of the poor’s asset portfolios or fundamental assets (see Table 2) (Moser & Satterthwaite 2008; Moser & Stein 2011; Simatele & Simatele 2015). This is partly because the AVAF is a diagnostic tool for understanding the factors responsible for the erosion of the assets of an individual.

**TABLE 2:** Definition of the five fundamental assets or capital for individuals, households and communities.

Asset or capital	Definition
Physical	This includes equipment, infrastructure such as road networks and other productive resources owned by individuals, households, communities or the country itself.
Financial	This refers to financial resources available and easily accessible to individuals, which includes loans, access to credits and savings in a bank or any other financial institution.
Human	This refers to the level of education, skills, health status and nutrition of individuals. Labour is closely associated with human capital investments. Health statuses of individuals impact either positively or negatively on their ability to work, while skill and level of education is crucial because it influences individuals return from labour.
Social	This refers to the norms, rules, obligations, mutuality and trust embedded in social relations, social structures and societies’ institutional disposition.
Natural	This refers to the atmosphere, land, minerals, forests, water and wetlands. For the rural poor, land is an essential asset.

*Sources*: Bebbington, A., 1999, ‘Capitals and capabilities: A framework for analysing peasant viability, rural livelihoods and poverty’, *World Development* 27, 2021–2044. https://doi.org/10.1016/S0305-750X(99)00104-7; Moser, C., 2011, *A conceptual and operational framework for pro-poor asset adaptation to urban climate change*, viewed 24 October 2016, from https://pdfs.semanticscholar.org/04b1/bcdba119831cd40f0dde1073a5e9dd32a284.pdf; Moser, C. & Satterthwaite, D., 2008, *Towards pro-poor adaptation to climate change in the urban centres of low and middle income countries*, viewed 07 June 2018, from http://pubs.iied.org/pdfs/10564IIED.pdf; Thornton, P.K., Jones, P.G., Owiyo, T., Kruska, R.L., Herrero, M., Orindi, V. et al., 2008, ‘Climate change and poverty in Africa: Mapping hotspots of vulnerability’, *African Journal of Agricultural and Resource Economics* 2, 24–44.

Notwithstanding, having a comprehensive picture of the complexities and interconnectedness of factors that undermine the poor’s ability to maximise their bundle of assets during food production is equally essential. This is against the backdrop of the sustainable development goals (SDGs) – leaving no one behind – which emphasises, among other issues, the urgent need to meet the nutritional requirements of the poor. Thus, this will enable practitioners to identify the appropriate leverage points to inject decisive policy interventions aimed at significantly minimising the adverse effects of both climatic and non-climatic stressors, which can prevent the poor from maximising their asset portfolios during food production. In this regard, systems thinking (ST) can provide development practitioners with a useful lens to effectively identify the factors that impede the rural poor’s ability to maximise their asset portfolios during food production (Bradbury 2003; Kunsch, Theys & Brans 2007; Nguyen & Bosch 2013).

Against this background, we make a case for the AVAF and ST as an integrated methodological framework. We argue that the utilisation of this integrated methodological framework is pivotal to highlight the factors that compromise the poor’s ability to efficiently maximise their asset portfolios in pursuant of their livelihoods.

## Methodologies used to highlight climate change vulnerability in developing countries

As highlighted earlier, various participatory methodologies have been employed by researchers over the past two decades in order to meticulously unpack the factors that compromise the poor’s ability to produce their food efficiently (Chambers 1994; Ibarraran et al. 2010; Preston et al. 2011). While the various methodologies have proved useful in illuminating issues undermining effective food production, the search for more nuanced methodologies to effectively address the increasing poverty and inequalities besieging countries in SSA may implicitly reflect practitioners’ dissatisfaction with pre-existing methodologies (Richards 1995; Stadler 1995). In this regard, Moser and Stein (2011) contend that the use of AVAF will enable development practitioners to identify the factors that impede the poor’s ability to maximise their fundamental assets when relentlessly striving to obtain their livelihood. The advocacy for the use of AVAF is underpinned by the fact that the poor have to devise strategies to navigate the adverse impacts of climate change during food production, while simultaneously being faced with economic, social and political constraints (Moser & Satterthwaite 2008). This view is substantiated by Moser and Stein (2011) who argued that the poor’s inability to effectively engage in their livelihood practices is because of exposure to multidimensional risk that stems from environmental, social, political and economic factors. Thus, the utilisation of the AVAF will enable development practitioners to effectively capture the ways in which social, economic, political and environmental factors erode the asset portfolio of the poor and undermine their ability to engage more effectively in food production (Table 1). Consequently, this will arguably set in motion the process for development practitioners to carve out strategies aimed at protecting and strengthening the fundamental assets that are volatile to both climatic and non-climatic stressors (Moser & Stein 2011). This is crucial because by looking at poor’s asset portfolios, one can comprehensively deduce those that will be able to quickly bounce back into food production after being confronted by either climatic and non-climatic stressors (Prowse & Scott 2008). It is therefore not surprising to note that Ebhuoma and Simatele (2017a) have argued that until pro-poor policy interventions are geared towards strengthening the fundamental assets that play a pivotal role in the livelihood activities of the poor, the battle to outwit climatic impacts will remain a utopian fiction.

Nonetheless, the use of the AVAF alone, we argue, may not comprehensively underline the interconnectedness and the complex ways in which the factors limiting the poor from maximising their asset portfolios undermine effective food production. As in the Indian folklore, about six blind men who touched an elephant and reached contrasting conclusions about the meaning of the entire object according to the part they felt (Nguyen et al. 2011), factors responsible for depleting the asset portfolios of rural households may be interpreted differently by various development practitioners. Avoiding or significantly minimising the attribution of different meanings to factors that contribute to asset portfolios depletion will depend on development practitioners being equipped with a holistic understanding of the factors that could trigger the erosion of the poor’s asset portfolios in the communities they are responsible for. Against this background, we argue that ST can effectively complement the AVAF by providing a holistic understanding of the causal factors undermining the poor’s ability to maximise their asset portfolios during food production. As documented by Maani and Cavana (2007), ST offers a holistic way of appreciating all dimensions of a complex problem and enables the formation of effective long-term management strategies. Thus, the application of ST is crucial to identify leverage points where policy interventions can be injected in order to foster breakthrough outcomes, necessary to avert the inability for the poor to maximise their asset portfolios during food production.

In light of the above, the application of the integrated methodological framework – AVAF and ST – will enable development practitioners to effectively address issues that trigger both the erosion of the poor’s asset portfolios and the factors impeding effective food production practices at its root cause (see, e.g., Bradbury 2003; Koester, Eflin & Vann 2006). By tackling issues at its root cause, Bradbury (2003) and Koester et al. (2006) contend that development practitioners will be able to avoid formulating policies that could potentially result in maladaptation in the future.

## Materials and methods

### Snapshot of the study areas

**FIGURE 1:**
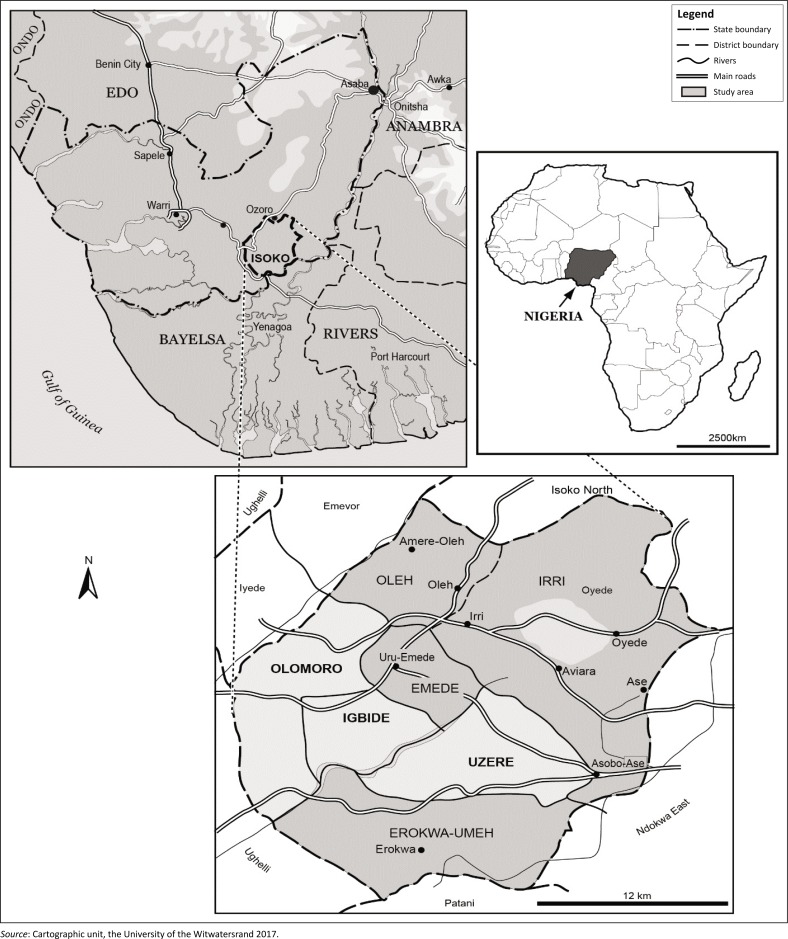
Map of the study areas in the Delta State, Nigeria.

The study was conducted in Olomoro, Igbide and Uzere communities in Isoko South Local Government Area (ISLGA) of the Delta State (Figure 1). These communities, which are predominantly agrarian, are homogenous in nature arguably because of proximity to one another. Firstly, they are all oil-producing communities. The three communities, en masse, play host to 37 oil wells where oil exploration activities were carried out by Shell Petroleum (a multinational oil company) for over four decades before selling the right of production to Integrated Data Services Limited (IDSL) in 2014 (Ebhuoma & Simatele 2017a). Secondly, they speak the same local dialect (*Isoko*) and rely on indigenous knowledge systems (IKS) to predict future weather conditions. It is noteworthy to mention that the farmers in all three communities produce the same major crops – cassava and groundnut – annually, with the women being the primary drivers of food production. The men, on the other hand, usually engage in fishing, although some assist their wives on the field.

The choice of these communities as the study areas, which are classified as coastal communities (Fabiyi & Oloukoi 2013), was hinged on the observation made by Omohode (2012) immediately after the 2012 flood. Omohode (2012) argued that majority of the coastal communities in ISLGA were completely submerged without a visible trace of any building. This made the region to appear like an emergency ocean when surveyed from a distance. Thus, the aforementioned communities will be crucial to effectively understand the challenges faced by farmers as they attempt to employ their asset portfolios in order to attain household food security in the face of both climatic (flooding) and non-climatic stressors (e.g. oil exploration activities). This is arguably because of stiff contestations that have occurred in the past between indigenes of these communities and Shell Petroleum, owing to grievances about the adverse impacts of oil exploration and exploitation activities on indigenes health and livelihood activities (Adusei 2015; Ikelegbe 2005).

The farmlands in Igbide and Uzere are generally low-lying, while Olomoro comprises both low- and high-lying farmlands. The mean annual rainfall from March to October in the Delta State ranges from 2500 to 3000 mm (Niger Delta Environmental Survey [NDES] 2000). The low-lying farmlands in each of the study areas are usually inundated from mid-June, at the earliest, to the last week in October every year. In extreme conditions, the low-lying farmlands remain inundated until the third week in November (Ebhuoma & Simatele 2017a). Thus, while other crops – groundnut, pepper, yam, cocoyam and plantain – which mature within 4 months are cultivated anytime between the last week in February and March, cassava, which requires a minimum of 6 months to attain maturity, is usually planted in December and harvested between June and August each year on the low-lying farmlands through a strategy indigenously referred to as *elelame* (follow-water-go).

*Elelame* is embarked upon anywhere between the first and second weeks in December, immediately after the seasonal floodwater starts retreating the farmlands disproportionately. Immediately, some portions of the soil become visible and moist, the farmers start cultivating. As the floodwater continues to recede in other portions of the farmlands, the farmers employ similar cultivation strategy.

At the commencement of the rainy season in the following year (normally in June), the floodwater starts inundating the low-lying farmlands disproportionately from mid-June, although some participants argued that it starts occurring in July in other farmlands. Just before the floodwater begins to inundate the low-lying farmlands, the farmers start harvesting the produce in proximity to the point where the farmlands are expected to be first inundated, to about 5 m – 20 m away (depending on the terrain of the farmland) from the anticipated starting point. The fundamental reason for employing this strategy is that in the eventuality of not being opportune to return to the farmland within the next 4–5 days, the floodwaters will not get to the point where they temporarily stopped harvesting. If, however, the floodwaters get to the point where they stopped harvesting, the cassavas submerged in the floodwaters can still be harvested without having gone rotten (Ebhuoma & Simatele 2017a). Insufficient household workforce is also a crucial determinant of the extent to which farmers temporarily stop harvesting. This is because some farmers may have about five plots of farmlands in different locations within the community where they planted cassava on.

### Methodology

The AVAF, which identifies the linkages between different vulnerabilities and the fundamental assets in the portfolio of the rural poor (Moser & Stein 2011), and ST were the methodological frameworks used in this study. Focus group discussions (FGDs) and semi-structured interviews advanced the use of the AVAF, which included Venn (or institutional) diagrams, transect walks, brainstorming, community risk mapping and historical timelines. The study comprised 35 FGDs and four one-to-one semi-structured interviews (two in Olomoro, one in Igbide and one in Uzere) conducted between June and October 2015 and July 2016 (see Table 3).

**TABLE 3:** Gender distribution of study participants.

Gender	Igbide		Uzere		Olomoro	
	Frequency	Percentage	Frequency	Percentage	Frequency	Percentage
Women	41	69	36	71	43	63
Men	18	31	15	29	25	37

**Total**	**59**	**100**	**51**	**100**	**68**	**100**

Each FGD was made up of 3–12 participants between the ages of 20 and 85. The age criterion was hinged on the fact that only adults could participate in the study because of the conditions of the approved ethical clearance. Eligible participants were identified by the help of key informants who have been residing in each of the communities for over 20 years, and an agricultural extension officer in ISLGA. Specific criteria used to select eligible participants for the study included those who have been farming in each of the study areas for a minimum of 10 years, as they are likely to have a vast wealth of experience regarding the ways climatic and non-climatic stressors impact negatively on food production. Others include gender, those whose household assets and livelihoods were adversely affected by the 2012 flood disaster, those who cultivate predominantly on low-lying farmlands and willingness to participate in the study. The qualitative data were analysed using the content thematic analysis technique. This analytical technique enabled the quantitative representations (in percentages) of participants’ responses to issues under investigation as highlighted in the ‘Results’ section, which emanated from both the FGDs and semi-structured interviews (see Rejnö, Berg & Danielson 2012).

### Ethical considerations

Approval to conduct the study was received from the Human Research Ethics Committee (Non-Medical) of the University of the Witwatersrand (R14/49 Ebhuoma).

## Results

### Asset vulnerability analytical framework as a diagnostic tool to highlight factors eroding farmers’ asset portfolios

The degradation of natural capital as well as the erosion of financial, human and physical capitals have made food production an uphill task in the study areas. In the context of natural capital, participants (92%) argue that because of the seasonal inundation of their low-lying farmlands, they are constrained to cultivate for approximately 8 months annually. For instance, a participant explained:

‘The problem with our farming system is the seasonal planting that we are constrained to undertake annually. We must harvest all our produce before our farmland becomes inundated. This usually exacerbates food insecurity when a household gets a poor harvest in a particular season … This is the advantage farmers in neighbouring communities, who cultivate on high ground, have over us. “They do not lack *garri* (processed cassava) at any point in time throughout the year”.’ (Male farmer from Uzere community, in his eighties)

As if the aforementioned is not already making it challenging for the participants to successfully transcend living below the global poverty index of less than $2.00 a day, they argued that the quality of food produced over the last two decades has declined abruptly because of ‘oil exploration-led exploitation’. In fact, most participants (89%) vehemently argued that oil exploration is responsible for the reduced quality of food produced in their respective communities. In this regard, a participant commented:

‘Prior to oil exploration activities in Olomoro, in the 1960’s, the *garri* we produced was known in the Delta State for its high starch content. However, community members began to observe a reduction of the starch content of the *garri* they produce from the 1980’s. Today, the *garri* produced in Olomoro has virtually no significant starch content.’ (Female farmer from Olomoro community, in her sixties)

The resultant effect of this, as argued by the farmers, is that consumers now prefer to purchase *garri* from neighbouring communities that are void of oil exploration activities because of its ‘higher starch content’. Along this continuum, a participant lamented:

‘There has been a significant reduction in the nutritional value of the *garri* we produce due to the oil exploration activities that have been on-going for over four decades…Before Shell started drilling oil from our community, we were promised that they would carry out remedial actions regularly to ensure that the soil does not lose its nutrients. Up till today, no remedial action has been carried in this community.’ (Female farmer from Igbide community, in her fifties)

However, not all participants were in total agreement with this line of thought. While acknowledging the deleterious effects that oil exploration activities have had on food production, a few participants (7%) emphasised that farmers’ inability to practise bush fallowing is also another major factor that has compromised the quality of food produced. For example, a participant explained:

‘Between the 1960s and 1980s, farmers could afford to leave their farmlands to go fallow for three to seven years before going back there to cultivate on it. Within the last two decades, however, the demand for arable farmlands has witnessed an abrupt increase. This is due to the sporadic increase in birth rate whereby it has become usual for a 15-year-old boy to have a partner and child. Consequently, the father of the 15-year-old boy has no choice but to give a piece of land to his son to enable him and his partner grow their food. This has made it almost impossible for some farmers to practice bush fallowing.’ (Male farmer from Olomoro community, in his fifties)

The overwhelming proliferation in population in each community, coupled with indigenes in diaspora’s quest to build themselves houses in their respective communities of origin, has also triggered the conversion of farmlands to secure shelter, thereby making arable farmlands a relatively scarce commodity. This has also compromised farmers’ ability in the study areas to practise bush fallowing.

In the context of financial capital, the participants lamented that irregularities and inconsistencies surrounded the disbursement of farm loans. All but one farmer, for example, claimed that they have never been beneficiaries of any agricultural loans. In this regard, a participant commented:

‘We only hear of farm loans and other incentives meant for farmers in this community after it has been disbursed. The only beneficiaries of farm loans are relatives of those working in the office responsible for disbursing farm loans. The painful part is that these relatives usually hoard the information to themselves during the loan distribution processes.’ (Female farmer from Uzere community, in her forties)

From a human capital perspective, physical and emotional abuse in Olomoro, in particular, was underlined as a factor that impedes women’s ability to engage effectively in food production, as it reduces their labour force drastically. A few explained that this has become a regular occurrence for some women in the community with their husbands as the chief perpetrators. When probed about the role of the police in attempting to cushion the plight of the women, a participant explained:

‘Do we have a police station in this community? When the men are arrested, with a bribe of ₦1000,00 [Nigerian naira] (approximately $3,00 US) they are released. I don’t know what we have in this community but it is certainly not a police station.’ (Female farmer from Olomoro, in her thirties)

With regard to physical capital, the farmers complained that despite the enormous contributions that their communities have made to the nation’s foreign revenue for over four decades, their communities have remained inconceivably underdeveloped. The lack of good road networks within each community, for example, usually erodes the financial capital of the farmers, albeit insidiously. To illustrate, during the rainy seasons, it can be excruciating for motorists to weave their way through the aforementioned communities. This is because of the numerous potholes that are usually engulfed with floodwaters, which makes it difficult particularly for those not familiar with the road to swiftly navigate their way through it. This makes access to markets, where they have to sell some of their farm produce, extremely difficult and expensive.

It is noteworthy to mention that both the government and Shell Petroleum have not been oblivious to the plight of the people. The challenge, however, is that whenever the government wants to fix the roads or Shell Petroleum wants to carry out some of its corporate social responsibility, for example, some affluent people in the Delta State will ensure that the contracts are awarded to their affiliates. After being awarded the contracts, most contractors often use substandard materials to carry out the required projects like road repairs, for example, which will not last longer as expected. The ability for most contractors to carry out substandard jobs with impunity was perceived by the participants as ineptitude on the part of government. This, among other issues, has amplified their grievances towards the government.

**FIGURE 2:**
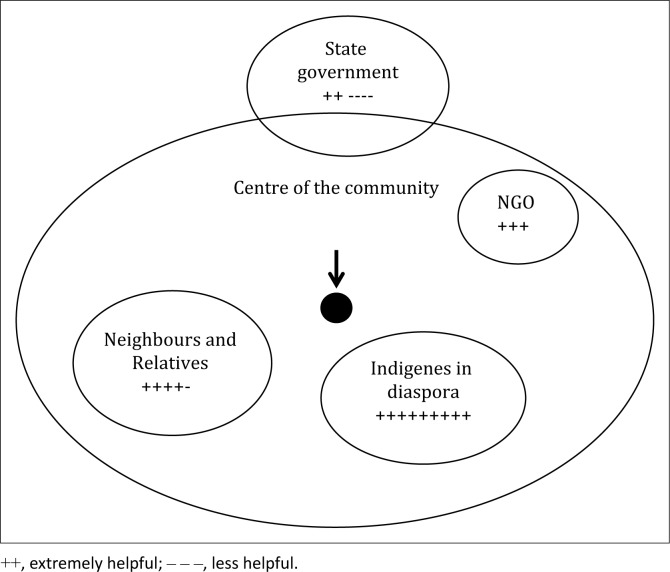
Venn diagram showing important institutions that enabled farmers bounce back into food production in the aftermath of the 2012 flood disaster.

It was also highlighted that for those privileged to have access to large areas of farmlands enough to engage in commercial farming, their inability to access farm machineries have continued to dampen their fight to transcend the boundaries of a subsistence farmer. A male participant in Uzere, for example, explained that while the Delta State government usually provides farm equipment for farmers, ‘it never gets to them’. The participant also complained that this equipment is ‘always hijacked’ by dignitaries and close associates of key politicians in the Delta State for their selfish interest. Also, 8% of the elderly participants (50 years and above) acknowledged that in the 1960s they used to produce rice. This is because of the swampy nature of their farmlands during the rainy season, and because the government provided rice milling machines to enable farmers process the produce. However, since the 1970s till date, no provision has been made to provide rice milling machines for farmers. Thus, rice cultivation has been abandoned. In view of the above, it was not surprising to observe that the participants felt neglected by the government in the aftermath of the 2012 flood (Figure 2).

The 2012 flood, classified as the most devastating in the history of Nigeria, agonisingly impacted Igbide, Uzere and Olomoro communities, which severely compromised households’ food and nutrition security for the next planting season (Ebhuoma & Simatele 2017a). While the participants acknowledged that the Delta State government made provision for items including stems to be distributed to the farmers, they lamented that most of the affected victims did not get anything. Instead, it was fadama^1^ development project, an initiative of the World Bank whose aim is to improve the general welfare of users of rural lands and water resources from 01 July 2008 to 31 December2019 (World Bank 2018), which provided seedlings and fertilisers for the affected farmers. Also, indigenes in the diaspora, individuals and relatives in neighbouring communities that were unaffected by the flood provided cassava stems for the affected farmers.

It is noteworthy to mention that in spite of the myriad challenges that the farmers have to contend with on an annual basis, they still manage to produce food annually. Because food production is interwoven in a complex web of political, social, environmental, economic and cultural factors (Banson, Nguyen & Bosch 2016), the AVAF alone, we argue, will not suffice in presenting a holistic description and the long-term implications of the farmers’ inability to maximise their asset portfolios during food production. In this regard, ST approach is pivotal to enable development practitioners to get a comprehensive picture of the various factors that hamper the rural poor from maximising their asset portfolios during food production (Banson et al. 2016).

### Systems thinking application to highlight factors impeding effective food production

**FIGURE 3:**
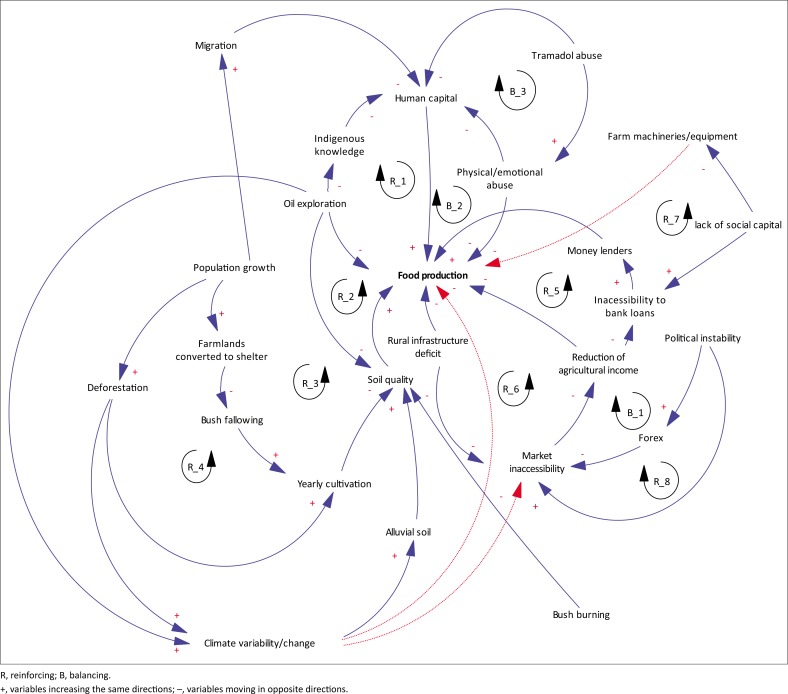
Systems model of factors undermining effective food production in the study areas. Dashed lines have no influence on the loop they pass through.

Causal loop modelling was used to produce an ST model to highlight the interconnections and interdependencies among the various components within Igbide, Uzere and Olomoro communities (Figure 3). Each variable can either progress in parallel or opposite directions. If an increase in one variable leads to a decrease in another variable, it is denoted by ‘-’ close to the arrow head. Conversely, if an increase in one variable results in an increase in another variable, it is denoted by ‘+’ close to the arrow head. The ST model, as argued by Kunsch et al. (2007) and Flood (2010), is analysed through feedback loops. Feedback loops are either balancing or reinforcing. Balancing loops seek to stabilise a system or return it back to normal. Reinforcing loops, on the other hand, usually represent increasing or decreasing actions (Nguyen & Bosch 2013). Figure 3 identified eight reinforcing (R) loops and three balancing (B) loops. For reasons of brevity, we have deliberated on issues that could eventually result in making food production overwhelmingly difficult within the shortest timeframe if decisive measures are not taken by development practitioners.

From Figure 3, a glaring issue that is impacting negatively food production is the abuse of the drug *tramadol*. It was argued that this has constituted a menace in these communities including the depletion of household workforce, thereby compromising the ability of affected households to produce more food. Some participants explained that while the sale of drug is illegal, some pharmaceuticals still make this drug readily available for those in need of the substance. It was alleged that one reason the drug is highly sought-after is that it ‘enhances stamina during sexual intercourse’. The adverse implications associated with a *tramadol* overdose by husbands or partners, which often results in some of them collapsing, are twofold: firstly, wives or partners are left with no alternative but to seek medical assistance for their husbands or partners. Even after their husbands or partners have been stabilised by a medical practitioner, the women still have to cater to their needs for some days as they need to be closely monitored at home. This ultimately results in disruption of food production and shortage of household labour in circumstances where the men assist their wives to produce food. This can have severe implications for households’ food security in circumstances where the households have not harvested all their cassava tubers on the low-lying farmlands before the seasonal inundation occurs (*elelame*), and are unable to return within the next 2 days to harvest the inundated tubers in order to prevent them from rotting away. Secondly, and overdose of *tramadol* by husbands or partners may cause emotional abuse in women (B_3) through shaming and mockery by community members because the affected women will likely be perceived as sexually insatiable by their husbands’ or partners’ natural energy levels. This has the potential to impede the affected women from producing food efficiently because emotional well-being is crucial for maximum productivity (Donald et al. 2005).

Figure 3 also reveals that the participants have, for decades, relied and continue to rely on IKS not limited to the observation of the flowering of specific trees, croaking of frogs and cloud observation (see Ebhuoma & Simatele 2017b). For example, a participant explained:

‘The use of IKS [indigenous knowledge systems] by farmers in this community to determine when to start growing our food is hinged on tradition. Although it has not been 100% accurate at all times, it has, for decades, proved extremely beneficial to those who respect tradition. When farmers obey instructions from nature, they get rewarded bountifully.’ (Male farmer from Olomoro, in his sixties)

The participants, however, pointed out that IKS has not been reliable as it used to be in previous decades. While 3% of the participants suggested that only God can determine the cause regarding the inconsistences of their IKS, 18% argued that this is the resultant effect of oil exploration activities. In narrating how oil exploration activities have affected IKS, it was argued that gas flaring has negatively impacted the rubber trees and cassava leaves, which they rely upon to predict the weather. This is because, occasionally, the rubber trees and cassava leaves do not flower at the time ‘they are naturally supposed to’. When this happens, the tendency for their predictions to be inaccurate becomes relatively high. Thus, oil exploration activities, from the participants’ perspective, have undermined their traditional way of life.

From a political perspective, political instability has, in part, emanated from the federal governments’ introduction of a policy in 2016 that allowed the currency to trade freely in a move to control the currency crisis. This resulted in a significant devaluation of the naira, with the naira exchanging in December 2016 at 305 naira (₦305.00) to 1 US dollar, as against 155 naira in November 2014 (₦155.00) (Nigerian Bulletin 2016; Tijani 2015). This adversely reduced the purchasing power of most end-users (B_1), which, in turn, reduces farmers’ financial capital. This is because Nigeria depends largely on foreign products (Vanguard 2015). Thus, the soaring of the US dollars heightened the prices of most goods in the country, thereby creating a ripple effect by affecting other sectors of the country. Also, the government’s inability to pay workers’ salaries regularly at the end of each month – in some instances, workers are owed over 6 months salaries (Vanguard 2018) – has significantly reduced the purchasing power for most consumers, with severe implications for the financial capital of the farmers (R_8). The lack of financial capital impedes farmers’ ability to purchase seedlings, fertilisers and other basic items necessary to produce food efficiently.

In the context of population growth, it was argued that it has facilitated the conversion of farmlands into housing units. Further, the increase in population, which also triggered deforestation, catalysed the inability for farmers to leave their farmlands to undergo bush fallowing as they usually did between the 1960s and the 1980s in order to produce ‘enough’ food to meet their livelihood objectives. Thus, the increase in population has made yearly food production in the limited available farmlands the norm. This has also contributed to the low quality of food produced because of a significant reduction in the soil quality (R_3). In the context of soil quality, the participants (89%) echoed that oil exploration activities remain the causal factor for the reduced soil quality (R_2); an elderly participant from Uzere, in his 80s, provided a mind-boggling insight. Not refuting the claim that oil exploration activities have compromised the soil quality, he argued that the annual seasonal flooding which occurs on the low-lying farmlands brings along with it alluvial soil. This, as he further pointed out, helps to replenish the soils’ lost nutrients. He, however, concluded that oil exploration activities have expedited the loss of soil nutrients at a pace faster than the rate at which alluvial soil replenishes the lost nutrients.

## Discussion

The use of participatory methodologies such as CCVR, PRA, CVCA, PVA, PIA and VM to conduct vulnerability assessments has undoubtedly revolutionised the ways social scientists conduct their research. Nonetheless, they have not explicitly highlighted how the multidimensional aspects of socio-economic factors facilitate the vulnerability of assets in the portfolio of the poor. While it is crucial to understand how the aforementioned factors facilitate asset vulnerability, development practitioners stand the risk of failing to get a comprehensive view of the ways this might lead to the increased difficulties of rural households to produce food efficiently in the future. Thus, as revealed in the results, ST serves to compensate for the deficit. Through the utilisation of the AVAF and ST as a twin methodology, findings from this study can be categorised into three major points.

Firstly, physical and emotional abuses impede rural women’s ability to produce food efficiently. Issues pertaining to physical and emotional abuse of rural women have been documented (Jewkes et al. 2001; United Nations [UN] 2012). The severity of physical and emotional abuse lies in its powers to deplete human capital, which is essential for efficient food production. In spite of these setbacks, they are often not given due consideration in deliberations regarding the ways to upscale food production in the rural Delta State. Instead, development practitioners place ‘huge emphases’ on how to ensure farmers have easy access to arable farmlands, farm loans and climate services (Ebhuoma & Simatele 2017b). Thus, without the utilisation of the AVAF and ST as a twin methodology, development practitioners may not get a holistic view about the severity of this seemingly trivialised but arguably serious bottleneck which compromises effective food production. We argue that the continual neglect of this delicate issue might suffocate the attainment of the second SDG, which aim is to attain zero hunger by 2030, among households in Igbide, Uzere and Olomoro because women are the primary drivers of food production.

Secondly, IKSs play a fundamental role in enabling farmers in the aforementioned communities to decide when and the quantity of food to produce in any farming season, even though IKSs have not being as accurate as it used to be in previous decades. This observation mirrors the findings of Roncoli, Ingram and Kirshen (2002), which suggest that IKS continues to play a fundamental role in dictating the affairs of rural food production despite its increased unreliability. While studies from Mexico (Eakin 1999), Burkina-Faso (Roncoli et al. 2002) and Malawi (Nkomwa et al. 2014) suggest that climate variability is the primary causal factor responsible for the increased inaccuracy of IKS, 18% of the participants in the current study argued that crude oil exploration activities are the culprit responsible for the increased unreliability of their IKS. Their views might be underpinned by the fact that there is evidence to suggest that the degradation of the natural asset in ISLGA, which has adversely affected the quality of food produced by the farmers, is largely because of oil exploration and exploitation activities (see Elum, Mopipi & Henri-Ukoha 2016; Ite et al. 2013). This has compounded participants’ inability to produce food efficiently because of loss of soil nutrients, although the inability to engage in bush fallowing was also acknowledged to contribute to the decline in quality of farm produce.

It is noteworthy to mention that the inability for most participants to mention bush fallowing as a factor contributing to a decline in quality of food produce may be hinged on the fact that they are bewildered and traumatised over the lack of development and rural infrastructures in their respective communities. This is, perhaps, because of the significant contributions of their communities – via crude oil exploration and exploitation activities for over four decades –to the nation’s foreign reserve (Ikelegbe 2005). The lack of development, rural infrastructure and environmental degradation through oil exploitation activities might be the resultant effect of a lack of social capital (see Figure 3), that is, because they lack indigenes from their communities that have held strategic positions, such as being high-ranking military officers or members of the National Assembly, in Nigeria.

The lack of development and rural infrastructure in the Niger Delta, as documented by Watts (2004), can be attributed to the institutionalisation of governance in Nigeria. For example, prior to the transition to democratic rule, in 1999, Nigeria was under military rule. The 1999 Constitution, which was formulated by ‘self-serving military officers’ many of whom were Northern ethnicity, led to the creation of more states in the North Western and North Eastern geopolitical zones of Nigeria than they deserve (PM News 2016). By creating more states and local government areas in the aforementioned regions, this favoured the military officers’ ethnicity in terms of distributing national revenue and left other geopolitical zones like the South-South, which the Niger Delta falls under, heavily disadvantaged (PM News 2016). This is possible because Nigerian law stipulates that any natural resources, including crude oil, belong to the federal government regardless of whose land the resources were discovered. This model of politicking in the Niger Delta paved the way for the systematic looting and sustained impoverishment of the Niger Delta through oil exploration and exploitation activities (Watts 2004), albeit the challenge of the Niger Delta was further amplified by mismanagement of revenue allocated to the region (PM News 2016). By utilising both the AVAF and ST frameworks, however, one can deduce that the lack of social capital might be the reason why environmental injustices, perpetuated by oil companies, have flourished because the government prioritises ‘economic development’ at the expense of natural capital conservation.

Thirdly, political instability has, in part, had a ripple effect on the farmers as the officials responsible for disbursing farm loans to farmers are government workers, who often go for months without being paid their salaries. Thus, despite the government availing farm loans for subsistence farmers in Nigeria for over two decades (Anyanwu 2004; Olaitan 2006; Philip et al. 2009), most farmers without social capital are hardly beneficiaries of government-approved farm loans as the information is usually ‘hoarded’ from them. It can be argued that this is because officials responsible for disbursing farm loans have become masters of the art of distributing the loans to close relatives and friends with impunity. However, for the fortunate farmers without social capital who become aware of the government-approved loans, officials responsible for disbursing the loans create unauthorised schemes not limited to coercing farmers to purchase items like seedlings and fertilisers, and thereafter, deducting the cumulative cost from the amount designated to each farmer as prerequisites for releasing the farm loans.

As farm loans can be crucial to enabling rural subsistence farmers in Nigeria engage efficiently in food production (Ebhuoma & Simatele 2017a), their inability to access government-regulated farm loans inadvertently means that rural subsistence farmers are left with no alternative but to secure loans from unregulated sources like money lenders. While obtaining loans from money lenders leads to increase in food production (see Figure 3, R_5), the drawback is that by the time the participants must have sold some of their farm produce to pay the loans, they are virtually left with nothing significant. This is because money lenders in Igbide, Uzere and Olomoro communities place as much as 40% interest rate on their loans for a 6-month period (Ebhuoma & Simatele 2017a). Thus, this model of food production will not enable rural households to evade living below the global poverty index of less than US$2.00 a day.

It is important to point out that issues relating to farmers’ inability to access government-approved loans and political instability are not peculiar to Nigeria alone. Parallel issues have been identified as a major bottleneck impeding the trickling down of resources that can significantly improve the welfare of farmers in other SSA countries (see, e.g. Bird & Shepherd 2003; Frost et al. 2007). However, various vulnerability studies that have employed participatory frameworks such as CCVR, PRA, CVCA and VM have, in some instances, not explicitly identified the dynamics in which these sorts of issues occur. For instance, in an attempt to address issues relating to farmers’ inability to secure government-approved farm loans, development practitioners might swiftly call for the retrenchment or replacement of corrupt officials with new transparent officers. While this interventional strategy may be apt, it fails to tackle the root cause of the problem, which stems from political instability. As documented by Nguyen et al. (2011), tackling societal issues without having a holistic view of how the entire system functions, including the underpinning factors responsible for the current state of affairs, will not yield the result as envisaged by the practitioners. Thus, the utilisation of the AVAF and ST, as an integrated methodological framework, will enable development practitioners, in collaboration with government officials, to come up with strategies that may provide lasting solutions to the problem.

In light of the above discussions and deliberations, it can be argued that the utilisation of AVAF and ST as an integrated methodological framework is pivotal to carry out contemporary vulnerability studies. However, it is important for researchers not to allow the euphoria of this integrated methodology overwhelm them to the point where they ignore DeWalt’s (1985) assertion. He contends that every new-found methodology initially undergoes a euphoric appeal among social scientists, to the stage of critical evaluation and debate about its usefulness in illuminating issues of vulnerability before it finally ends up in the buzz word ‘refuse dump’. Avoiding this dilemma will largely depend on highlighting its weaknesses. The integrated methodological framework, unfortunately, ignores the historical perspective of the ways in which legislations that govern the natural resources, for example, have contributed to the inability of rural households to currently obtain their livelihoods without certain difficulties.

## Conclusion

Various methodological frameworks have been developed in the last three decades in a bid to carve out the most ‘perfect’ method for conducting vulnerability assessments. Methodologies such as PRA, RRA PIA, VM, CCVR and CVCA have been utilised. However, these methodologies have not explicitly and holistically been able to highlight the factors that compromise farmers’ ability to produce food efficiently from an asset-based perspective. Thus, by utilising the AVAF and ST, this study highlights the factors facilitating the erosion of farmers’ asset portfolios in Igbide, Uzere and Olomoro, which undermine effective food production.

It is important to note that we do not portray the utilisation of AVAF and ST, as an integrated methodological framework, as the pinnacle of participatory methodologies when attempting to ascertain farmers’ vulnerability as it has its own shortcoming. Nonetheless, in a time when practitioners have recognised that poverty is multifaceted, creating avenues for the poor to accumulate assets is essential. Thus, the utilisation of the integrated methodology will make it relatively easier for development practitioners to identify the factors that undermine the poor’s ability to maximise their assets, including the long-term consequences of the continued inability of the poor to maximise their assets during food production. This will enable practitioners to implement the appropriate policy interventions aimed at protecting and strengthening the poor’s livelihood assets.

## Footnotes

1 ‘Fadama’ is an *Hausa* word, which is an indigenous language. It translates to a valley-bottom, flood plain or a lowland around a river that floods or becomes wet when the river is high.
